# Increased Bone Mineral Density with Monthly Intravenous Ibandronate Contributes to Fracture Risk Reduction in Patients with Primary Osteoporosis: Three-Year Analysis of the MOVER Study

**DOI:** 10.1007/s00223-014-9927-7

**Published:** 2014-11-07

**Authors:** Hiroshi Hagino, Seitaro Yoshida, Junko Hashimoto, Masayuki Matsunaga, Masato Tobinai, Toshitaka Nakamura

**Affiliations:** 1 School of Health Science & Rehabilitation Division, Tottori University Faculty of Medicine, Tottori, Japan; 2 Clinical Development Division, Chugai Pharmaceutical Co. Ltd., Tokyo, Japan; 3 Project & Lifecycle Management Unit, Chugai Pharmaceutical Co. Ltd., Tokyo, Japan; 4 Medical Affairs Division, Chugai Pharmaceutical Co. Ltd., Tokyo, Japan; 5 National Center for Global Health and Medicine, Tokyo, Japan

**Keywords:** Ibandronate, Vertebral fracture, Hip BMD change, Osteoporosis, MOVER study

## Abstract

The relationship between gains in bone mineral density (BMD) in the hip and the incidence of vertebral fractures in the MOVER study was examined. Japanese patients from the ibandronate and risedronate treatment groups whose hip BMD had increased during the 3-year treatment period were classified into those with or without vertebral fractures. In both the ibandronate group and the risedronate group, hip BMD gains in the patients who had developed no vertebral fractures during the treatment period were greater than in the patients who developed vertebral fractures. We categorized the gains in hip BMD at 6 months into 3 groups (≤0, >0 to ≤3, and >3 %), and used logistic regression analysis to estimate odds ratios and the probabilities of incidence of vertebral fractures at 12, 24, and 36 months. The current study demonstrated that greater gains in hip BMD during the first 6 months of treatment were associated with a reduction in the risk of subsequent vertebral fractures during the duration of treatment, and suggested that measurement of hip BMD gain at that time could lead to a prediction of the risk of the future vertebral fracture incidence.

## Introduction

In the treatment of osteoporosis, bisphosphonates have demonstrated their clinical efficacy, particularly their anti-fracture efficacy, and have become the most widely used anti-osteoporotic drugs worldwide. The anti-fracture efficacy of ibandronate was first demonstrated in the BONE study (oral iBandronate Osteoporosis vertebral fracture trial in North America and Europe) that examined treatment with oral ibandronate (2.5 mg/day and 20 mg intermittently) [1]. The MOBILE study (Monthly Oral iBandronate In LadiEs) was conducted to assess gains in bone mineral density (BMD) with oral ibandronate (100 and 150 mg/month) [2]. The DIVA study (Dosing IntraVenous Administration) was also conducted to assess gains in BMD with quarterly intravenous (IV) ibandronate injection (3 mg/3 months)  [3]. Analysis of pooled data from the MOBILE and DIVA trials showed that, for all clinical fractures, non-vertebral fractures, and clinical vertebral fractures, there was a significantly longer time to fracture events in the ibandronate group than in the placebo group over 5 years  [4]. Meta-analyses of the clinical studies also demonstrated that ibandronate had significant efficacy with respect to the risk reduction of not only key non-vertebral fractures but also of all non-vertebral fractures and clinical fractures [5, 6].

The relationship between increases in BMD and fracture risk reduction has been examined in meta-analyses of ibandronate. The increase in BMD in the lumbar spine over 2 years showed a reverse relationship with the incidence of clinical fractures, and the increase in hip BMD was associated with a reduction in the risk of non-vertebral fractures [7]. Gains in hip and lumbar spine BMD were also associated with a reduction in vertebral fracture risk, explaining a substantial proportion of the anti-fracture effect of ibandronate (23–37 % at 2 and 3 years) [8]. In fact, it has been reported elsewhere that decreased BMD is strongly associated with increased fracture risks and that increased BMD is predictive of the anti-fracture efficacy of treatment [9].

The MOVER study (MOnthly intraVenous ibandronatE versus daily oral Risedronate) was conducted for registration purposes in Japan [10]. The primary endpoint was the non-inferiority of ibandronate versus risedronate to prove its efficacy with respect to the incidence of non-traumatic vertebral fractures at 3 years. Since the anti-fracture efficacy of risedronate had been already demonstrated in randomized comparative studies  [11–13], risedronate was selected as a suitable active comparator in the MOVER study. The incidence rate of first new or worsening vertebral fractures was 16.1 % in the IV ibandronate 1 mg/month treatment group and 17.6 % in the oral risedronate 2.5 mg/day treatment group at 3 years. The hazard ratio of IV ibandronate 1 mg against risedronate was 0.88 (95 % confidence interval [CI] 0.61–1.27). Significant increases in BMD were observed in ibandronate 1 mg group as compared to the risedronate group. Based on the efficacy and safety data, monthly IV ibandronate 1 mg was approved for the treatment of osteoporosis in Japan. The current analysis from the MOVER study was conducted to examine the relationship between changes in hip BMD and the incidence of vertebral fractures, especially the impact that initial BMD gains have on the future incidence of vertebral fractures.

## Materials and Methods

The design of the MOVER study is already described [10]. Briefly, 1,265 patients with primary osteoporosis diagnosed according to the Diagnosis Criteria of Primary Osteoporosis in Japan [14] were randomized. The per-protocol population for the endpoint analysis comprised 376 patients in the ibandronate 0.5 mg group, 382 in the ibandronate 1 mg group, and 376 in the risedronate group (totally 1,134 patients). Baseline patient characteristics were well balanced between the treatment groups (Table 1). Morphometric vertebral fractures were assessed using semiquantitative methodology and quantitative morphometry by a central committee. BMD in lumbar spine (L2–L4) and total hip was centrally measured at baseline, 6, 12, 24, and 36 months using dual-energy X-ray absorptiometry (DXA) of Hologic and Lunar bone densitometers. In the current study, the patients in the ibandronate 1 mg group and the risedronate group were sorted by whether or not they developed vertebral fractures over the 3 years of treatment. The increase in BMD in the hip during the treatment period was examined to characterize its correlation with the anti-vertebral fracture efficacy of the drugs. Estimation of odds ratios and probabilities of developing vertebral fractures were performed based on logistic regression analysis. A separate logistic regression analysis was conducted for each of the treatment groups, and the dependent variable was the occurrence of fractures (with fractures) or non-occurrence of fractures (without fractures) during each of the treatment periods, while the independent variables included the change in hip BMD at 6 months (≤0, >0 to ≤3, or >3 %), number of existing fractures at screening (1 vs. ≥2 fractures), age at baseline (<75 vs. ≥75 years), change in adjusted urinary C-telopeptide levels at 6 months, and change in bone-specific alkaline phosphatase levels also at 6 months. Using the estimated regression parameters and the data of the individual subjects considered for the estimation of those parameters, the probability of fractures in each subject was calculated. This analysis was performed using SAS Version 9.2 (SAS Institute Inc., Cary, NC, USA).

**Table 1:** Baseline patient characteristics

	Ibandronate 0.5 mg (*n* = 376)	Ibandronate 1 mg (*n* = 382)	Risedronate (*n* = 376)
Women, *n* (%)	356 (94.7)	354 (92.7)	343 (91.2)
Mean age, years (SD)	72.9 (6.34)	72.2 (6.38)	73.0 (6.29)
Aged 60–74 years, *n* (%)	219 (58.2)	245 (64.1)	227 (60.4)
Aged ≥ 75 years, *n* (%)	157 (41.8)	137 (35.9)	149 (39.6)
Mean weight, kg (SD)	50.6 (8.00)	50.9 (7.36)	51.1 (8.35)
Mean height, cm (SD)	149.2 (6.66)	149.5 (6.56)	149.4 (6.70)
Mean BMD T-score (SD)			
Lumbar spine (L2–L4)	−2.71 (1.01)	−2.68 (1.01)	−2.59 (1.06)
Femoral neck	−2.48 (0.73)	−2.41 (0.80)	−2.53 (0.79)
Total hip	−2.17 (0.87)	−2.09 (0.86)	−2.18 (0.86)
Prevalent vertebral fractures, *n* (%)			
1	186 (49.5)	184 (48.2)	183 (48.7)
2	97 (25.8)	106 (27.7)	95 (25.3)
>2	93 (24.7)	92 (24.1)	98 (26.1)
Mean uCTX, µg/mmol CR (SD)	382.4 (226.2)	368.6 (209.9)	373.2 (261.0)
Mean uNTX, nM BCE/mM CR (SD)	73.6 (39.31)	69.4 (35.42)	68.9 (35.16)
Mean BALP, IU/L (SD)	33.6 (13.15)	33.9 (13.11)	32.4 (11.96)
Mean 25-OH vitamin D, ng/mL (SD)	19.6 (6.44)	20.0 (6.69)	19.7 (6.56)

*BALP* bone-specific alkaline phosphatase, *BCE* bovine collagen equivalent, *BMD* bone mineral density, *CR* creatinine, *SD* standard deviation, *uCTX* creatinine-corrected urinary collagen type 1 cross-linked C-telopeptide, *uNTX* creatinine-corrected urinary collagen type 1 cross-linked N-telopeptide

## Results

### Increase in hip BMD in Patients Without/With Vertebral Fractures

**Fig. 1:**
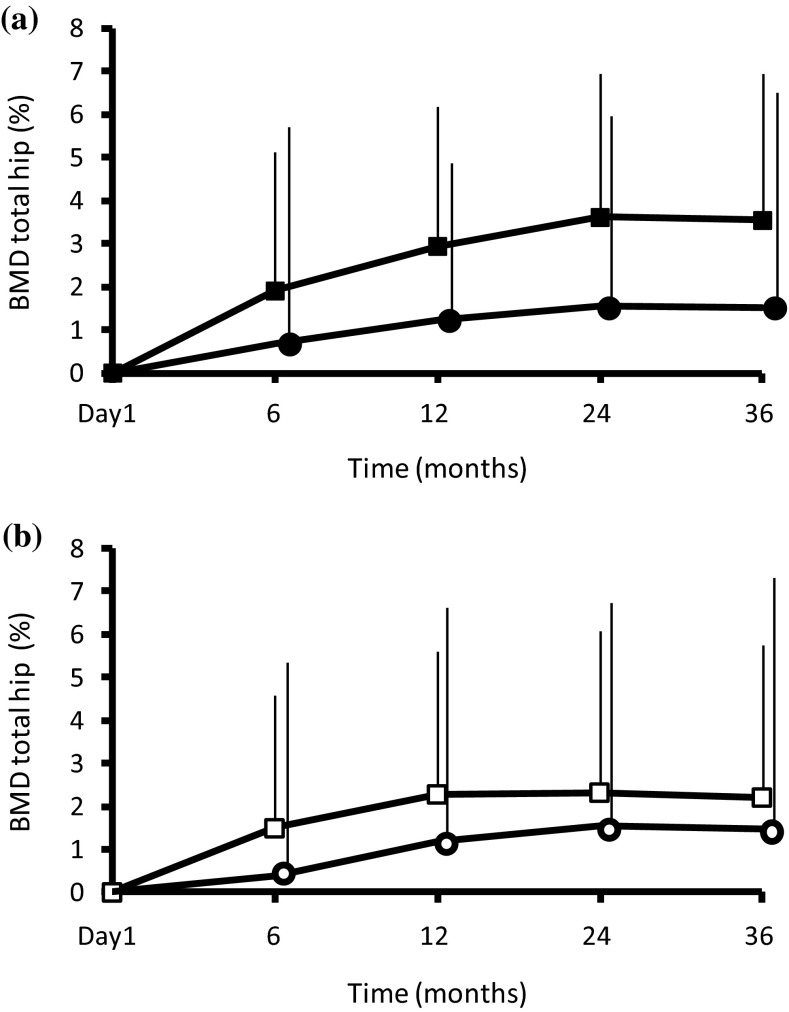
Mean increases (+SD) in total hip BMD in (**a**) ibandronate-treated patients with (*Black circle*) or without (*Black square*) vertebral fractures; (**b**) risedronate-treated patients with (*White circle*) or without (*White square*) vertebral fractures

The mean gains in BMD at 6 months relative to baseline in the ibandronate 1 mg and risedronate treatment groups were 1.7 and 1.3 % in the hip and 5.1 and 3.9 % in the lumbar spine, respectively, in the MOVER study [10]. Among patients receiving IV ibandronate 1 mg, the mean changes in hip BMD (Fig. 1a) in the group without vertebral fractures were 1.9 ± 3.2 % (mean ± SD) (*n* = 305) at 6 months, 2.9 ± 3.3 % (*n* = 290) at 1 year, 3.6 ± 3.3 % (*n* = 265) at 2 years, and 3.5 ± 3.4 % (*n* = 242) at 3 years. Among patients receiving IV ibandronate 1 mg, the mean changes in hip BMD in the group with vertebral fractures were 0.7 ± 4.9 % (*n* = 48) at 6 months, 1.2 ± 3.7 % (*n* = 47) at 1 year, 1.5 ± 4.4 % (*n* = 42) at 2 years, and 1.5 ± 5.0 % (*n* = 37) at 3 years. Among patients receiving oral risedronate 2.5 mg, the mean changes in hip BMD (Fig. 1b) in the group without vertebral fractures were 1.5 ± 3.1 % (*n* = 290) at 6 months, 2.3 ± 3.3 % (*n* = 276) at 1 year, 2.3 ± 3.7 % (*n* = 251) at 2 years, and 2.2 ± 3.5 % (*n* = 228) at 3 years. Among patients receiving oral risedronate 2.5 mg, the mean changes in hip BMD in the group with vertebral fractures were 0.4 ± 4.9 % (*n* = 60) at 6 months, 1.2 ± 5.4 % (*n* = 59) at 1 year, 1.5 ± 5.3 % (*n* = 53) at 2 years, and 1.5 ± 5.9 % (*n* = 49) at 3 years. In both treatment groups, the increases in hip BMD were substantially greater at all measurement times in the groups without vertebral fractures than in the groups with fractures.

### Future Vertebral Fracture Incidence Predicted by Changes in Hip BMD at 6 months

**Fig. 2:**
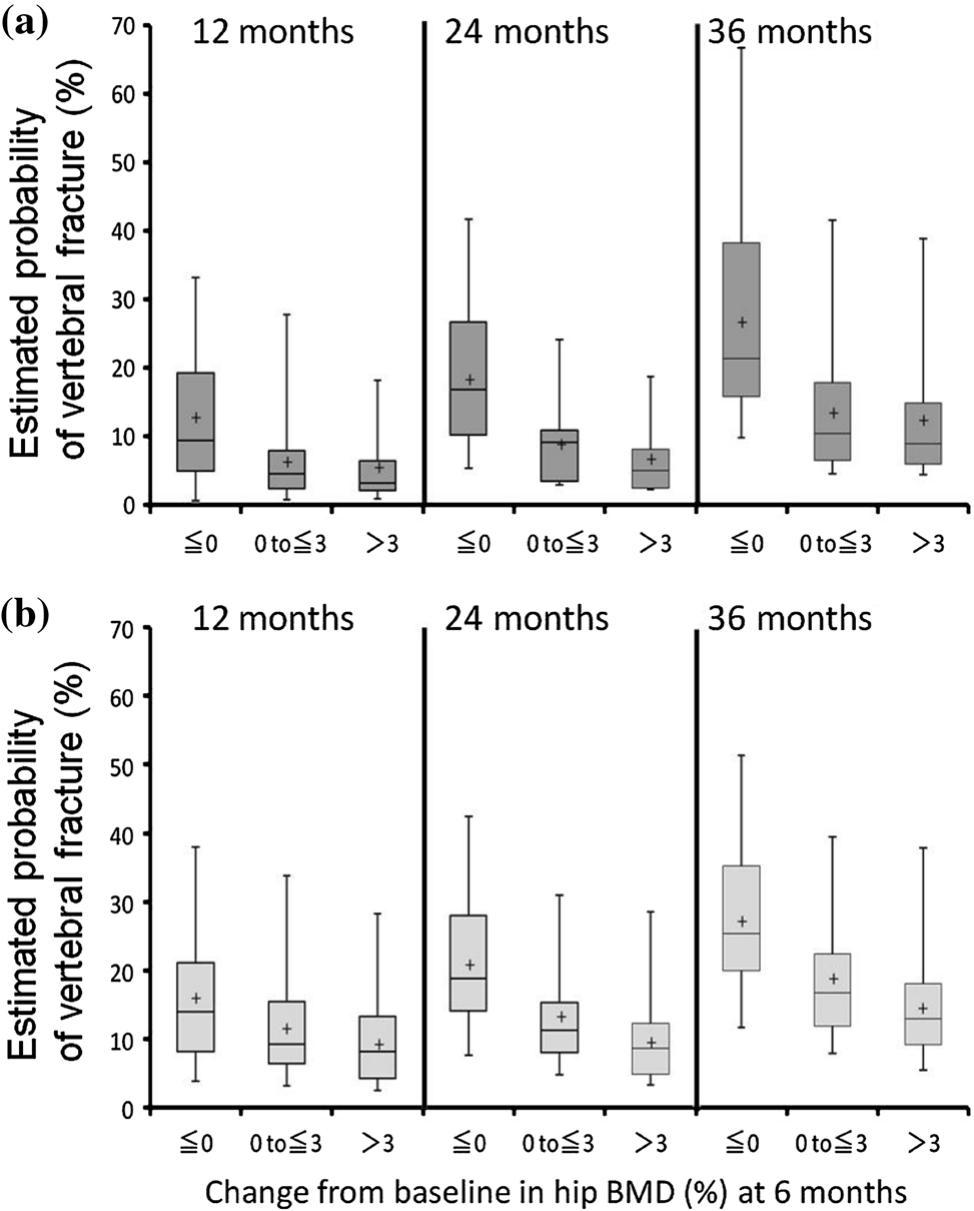
Estimated probability of incidence of vertebral fractures according to gains in hip BMD in patients treated with ibandronate (**a**) and risedronate (**b**) at 12 months (*left column*), 24 months (*middle*), and 36 months (*right*). The upper and lower fences represent the maximum and minimum values, respectively. The box represents the interquartile range. The cross indicates the mean value and the *horizontal line* indicates the median value

The gains in hip BMD at 6 months were categorized into 3 groups (≤0, >0 to ≤3, and >3 %). The probability of incidence of vertebral fractures in these 3 groups was estimated at 12, 24, and 36 months (Fig. 2a, ibandronate; Fig. 2b, risedronate). In the ibandronate group, the median probabilities of developing vertebral fractures at 12 months in each of the 3 groups were 9.5 % (*n* = 80), 4.6 % (*n* = 144), and 3.2 % (*n* = 114), respectively. In the risedronate group, the median probabilities of developing vertebral fractures at 12 months were 14.0 % (*n* = 113), 9.2 % (*n* = 138), and 8.2 % (*n* = 86), respectively. The median probabilities of developing vertebral fractures at 24 months were 16.8 % (*n* = 77), 9.2 % (*n* = 136), and 5.1 % (*n* = 109) in the 3 ibandronate groups and 18.8 % (*n* = 111), 11.2 % (*n* = 133), and 8.6 % (*n* = 85) in the 3 risedronate groups, respectively. The median probabilities of developing vertebral fractures at 36 months were 21.4 % (*n* = 72), 10.5 % (*n* = 128), and 8.9 % (*n* = 102) in the 3 ibandronate groups and 25.3 % (*n* = 100), 16.7 % (*n* = 119), and 13.0 % (*n* = 78) in the 3 risedronate groups, respectively. The numbers of vertebral fracture events that actually occurred (Table 2) were comparable to the probabilities of future vertebral fractures estimated by the logistic regression analysis.

**Table 2:** Numbers of vertebral fracture events according to change in hip BMD at 6 months

Treatment	Ibandronate			Risedronate		
	Hip BMD at 6 months					
	≤0 %	>0 to ≤3 %	>3 %	≤0%	>0 to ≤3 %	>3 %
(Months)	(*n* = 85)	(*n* = 150)	(*n* = 118)	(*n* = 116)	(*n* = 145)	(*n* = 89)
12	10 (12.5 %)	9 (6.3 %)	6 (5.3 %)	18 (15.9 %)	16 (11.6 %)	8 (9.3 %)
24	14 (18.2 %)	12 (8.8 %)	7 (6.4 %)	23 (20.7 %)	17 (12.8 %)	8 (9.4 %)
36	19 (26.4 %)	17 (13.3 %)	12 (11.8 %)	27 (27.0 %)	22 (18.5 %)	11 (14.1 %)

Treatment	Ibandronate			Risedronate		
	Lumbar spine BMD at 6 months^a^					
	≤0 %	>0 to ≤3 %	>3 %	≤0 %	>0 to ≤3 %	>3 %
(Months)	(*n* = 44)	(*n* = 81)	(*n* = 236)	(*n* = 61)	(*n* = 100)	(*n* = 190)
12	3 (7.7 %)	5 (6.3 %)	11 (4.8 %)	3 (5.2 %)	10 (10.3 %)	17 (9.3 %)
24	5 (12.8 %)	7 (9.2 %)	15 (7.0 %)	5 (9.1 %)	10 (10.6 %)	21 (11.7 %)
36	6 (17.1 %)	15 (22.4 %)	23 (11.2 %)	5 (10.0 %)	11 (12.8 %)	32 (20.3 %)

^a^Cases in which vertebral fractures occurred within the first 6 months were eliminated

The odds ratios for each group with a positive BMD response (>0 to ≤3 % increase from baseline, and >3 % increase) against the group with inadequate BMD response (≤0%) were compared for BMD gains in the hip and in the lumbar spine in the first 6 months of treatment (Table 3). With respect to the BMD gains in the hip, although the ratios were mostly numerically lower in the ibandronate group than in the risedronate group, it was shown that the BMD gains in the hip at 6 months promised similarly effective reduction in the risk of future vertebral fracture. The gains in BMD in the femoral neck showed similar trends in reduction of risk of future vertebral fracture (data not shown). With respect to the BMD gains in the lumbar spine at 6 months, the odds ratios were higher in the risedronate group than in the ibandronate group.

**Table 3:** Odds ratios of vertebral fracture incidence at 12, 24, or 36 months according to gains in hip or lumbar spine BMD at 6 months by treatment with ibandronate and risedronate

Gain in hip BMD at 6 months	Treatment duration	Against ≤0	Odds ratio (95 % CI)	
			Ibandronate	Risedronate
	12 months	>0 to ≤3 %	0.52 (0.20, 1.41)	0.79 (0.37, 1.70)
		>3 %	0.40 (0.13, 1.21)	0.54 (0.22, 1.36)
	24 months	>0 to ≤3 %	0.55 (0.23, 1.31)	0.64 (0.32, 1.31)
		>3 %	0.41 (0.15, 1.14)	0.41 (0.17, 1.00)
	36 months	>0 to ≤3 %	0.51 (0.24, 1.09)	0.69 (0.35, 1.33)
		>3 %	0.47 (0.20, 1.09)	0.44 (0.20, 0.99)

Gain in lumbar spine BMD at 6 months^a^	Treatment duration	Against ≤0	Odds ratio (95 % CI)	
			Ibandronate	Risedronate
	12 months	>0 to ≤3 %	0.87 (0.18, 4.16)	2.65 (0.67, 10.46)
		>3 %	0.77 (0.18, 3.21)	2.31 (0.62, 8.65)
	24 months	>0 to ≤3 %	0.76 (0.20, 2.79)	1.46 (0.46, 4.68)
		>3 %	0.70 (0.21, 2.29)	1.68 (0.57, 4.96)
	36 months	>0 to ≤3 %	1.54 (0.51, 4.63)	1.65 (0.52, 5.25)
		>3 %	0.72 (0.25, 2.03)	3.01 (1.05, 8.64)

^a^Cases in which vertebral fractures occurred within the first 6 months were eliminated

## Discussion

The purpose of this analysis of the MOVER study was to examine the relationship between gains in BMD and the occurrence of vertebral fractures by analyzing the gains in hip BMD in the initial 6 months and the subsequent development of vertebral fractures over time.

First, we compared the hip BMD gains in ibandronate- or risedronate-treated patients who developed vertebral fractures with those who had not developed vertebral fractures during the 3 years of treatment. In both the ibandronate and the risedronate treatment groups, hip BMD gains were greater in the patients who developed no vertebral fractures during the treatment period than in the patients who developed vertebral fractures. The hip BMD gains in the fracture-negative ibandronate group were consistently greater than in the fracture-negative risedronate group. On the other hand, both fracture-positive groups showed similarly low BMD gains. These results suggested that hip BMD gains could be an effective parameter with which to predict the future incidence of vertebral fractures.

Next, we categorized the patients into 3 groups according to the gains in hip BMD in the first 6 months of treatment, and we used logistic regression analysis to estimate future vertebral fracture risk reduction at 12, 24, and 36 months. Greater gains in hip BMD at 6 months were associated with a greater reduction in the risk of subsequent non-traumatic vertebral fractures over the 3-year treatment period. The BMD gains in the hip at 6 months, which might be the time-point when the effects of bisphosphonates begin to be seen [15], were shown in our analysis to predict the risk of vertebral fracture incidence. In all treatment periods, the probability of developing vertebral fractures tended to be lower in groups that had hip BMD gains of greater than 3 % than in those that had lesser gains in hip BMD. The numbers of fracture events also decreased according to the gain in BMD. The odds ratios in the hip BMD gains were low overall—under 1 over the 3 years—and the values decreased according to the BMD gains in the both treatment groups. Those results might explain why hip BMD gains might predict the future risk reduction of vertebral fracture. With respect to the BMD gains in the lumbar spine at 6 months, the odds ratios in the risedronate group were higher (but not significantly higher) than in the ibandronate group. It is recently reported that a bisphosphonate which has a lower mineral binding affinity such as ibandronate or risedronate could be efficiently delivered to the cortical bone area. It might be one of explanations that ibandronate showed the greater gains of BMD in the hip in a short period of 6 months [16, 17]. It has been also reported that the changes in lumbar spine BMD by treatment with risedronate contributed only 18 % (95 % CI 10, 26 %) of its efficacy against vertebral fractures [18]. The number of vertebral fracture events in the risedronate group (>0 to ≤3, and >3 % lumbar spine BMD increase) was greater than in the ibandronate group. Those might be a part of the reasons why the odds ratios in the risedronate group were over 1. However, further analysis of the individual cases is needed.

Many reports have said that BMD might be a factor predictive of future fractures in general. Additionally, when the BMD measurements of the lumbar spine, femoral neck, femoral trochanter, and hip were compared, hip BMD was reported to be better at predicting future incidence of all fractures than was lumbar spine BMD [19]. BMD measurements in all sites including hip were performed centrally at each time using DXA machine because the MOVER study was a randomized multicenter study. We examined the mean BMD values of each treatment group in the study, however, least significant change or coefficient of variation is important on BMD measurement. In clinical practice, due to variability of hip BMD measurement, there might be a difficulty to obtain the accurate data even though the treatment would express the efficacy at 6 months. The relationship between BMD in the first 6 months and the time-course of incidence of hip fractures should also be investigated; however, we did not get any information due to the low number of non-vertebral fractures including hip that occurred in the MOVER study.

Our analysis did not show the contribution rate of BMD gains to the expression of the anti-fracture efficacy in the MOVER study. It has been reported previously [8] that BMD gains by ibandronate would explain approximately one-third (24–37 %) of ibandronate’s anti-fracture efficacy. That contribution rate was derived from analysis of the BONE and IV studies in which the annual cumulative exposure (ACE) was under 5.5 mg. However, the ACE in the MOVER study was 12 mg; thus, the contribution of BMD gains in the MOVER study might be much bigger. We intend to calculate the contribution of BMD gains in future analysis.

Our current analysis indicated that greater gains of BMD in the hip in a relatively short period of 6 months of treatment were associated with a greater reduction in the risk of future vertebral fracture incidence. In fact, the BMD measurements using DXA machine are reimbursable every 4 month in Japan and the physicians like to measure BMD to evaluate the treatment regimen. We, in Japan, suggest to measure BMD after 6 months of therapy and keep to do every 6 month. In case the BMD measurement is not available or practical, the measurement of bone turnover markers (BTMs) could be supportive. In these days, not only physicians but also patients would discuss their laboratory data including BMD or BTMs values together, which is desirable to keep better adherence to therapy for osteoporosis. If there is no gain in BMD, it is an opportunity to re-assess the current therapy. To change drug would be one of next options and to add another drug would be also an alternative. The results suggest that the hip BMD value at 6-month treatment might be a useful predictor to prevent the future vertebral fracture incidence and provide an opportunity to assess the treatment options.
